# Epidural Catheter Bacterial Colonization and Infection in a Secondary Portuguese Hospital Setting

**DOI:** 10.7759/cureus.72428

**Published:** 2024-10-26

**Authors:** Catia Domingues, Luís Gonçalves, Marta Laranjo, Tiffany Santos, Décia Gonçalves, Lucia Gonçalves, Ana Cristina Barros, Gina Marrão, Ricardo Castro, Elisabete Valente

**Affiliations:** 1 Anesthesiology, Unidade Local de Saúde da Região de Leiria, Leiria, PRT; 2 Pathology, Unidade Local de Saúde da Região de Leiria, Leiria, PRT

**Keywords:** antibiotic resistance, epidural analgesia, epidural anesthesia, epidural space, hospital-acquired infections

## Abstract

Background and study goal

Epidural analgesia is effective for postoperative pain control but carries risks of infection, including bacterial colonization. This study aimed to determine the incidence of epidural catheter colonization and infection in a Portuguese hospital, identify common microorganisms and antibiotic resistance profiles, and assess potential risk factors.

Materials and methods

We conducted a prospective observational study from November 2022 to March 2023 in a Portuguese secondary care center. Adult patients undergoing surgery with epidural analgesia were included. Data related to patient demographics, surgical details, and the epidural technique were collected. Incidence rates were calculated, and risk factors for colonization were analyzed.

Results and discussion

Overall, 215 patients were included (mean age: 52.12 years; 76.7% women). The average catheter duration was 2.66 days. The incidence of bacterial colonization was 16.3% (95% CI=11.3-21.2%), with 74.3% showing antibiotic resistance. *Staphylococcus epidermidis* was the most frequent isolate (37.1%). No cases of epidural space infection were observed. Exploratory analyses indicated a potential higher risk of colonization when multiple punctures were needed (adjusted odds ratio (aOR): 4.52, p=0.33).

Conclusion

Epidural catheter colonization occurred in 16.3% of cases, with *Staphylococcus epidermidis* being the predominant agent. Despite colonization, clinical infections were rare. The high rate of antibiotic resistance underscores the need for improved stewardship and aseptic practices.

## Introduction

Epidural analgesia is a well-established technique for effective postoperative pain control, especially in major surgeries, due to its ability to reduce the stress response and improve outcomes [1,2]. However, despite its benefits, neuraxial anesthesia is not without complications, which may include epidural infections, meningitis, and skin infections at the puncture site [1,2].

Although routine aseptic precautions are taken during the placement of epidural catheters, international studies report colonization rates up to 28.8%, but no studies have examined the incidence of epidural catheter colonization in Portugal [3-9]. The primary route of colonization is often attributed to the patient's skin flora at the catheter insertion site, with *Staphylococcus epidermidis *being the most frequently identified microorganism [3,4,6,8,9]. Other potential routes include migration along the catheter tract and colonization during catheter handling or infusate administration, with colonization rates varying depending on antiseptic practices used, patient comorbidities, and the hospital environment in which the catheter is managed [3-5].

In this study, we aimed to determine the incidence of bacterial colonization and infection of epidural catheters in postoperative patients, identify associated microorganisms and their antibiotic resistance profiles, and assess risk factors contributing to colonization and infection, specifically within the Portuguese hospital setting, to evaluate the impact of local practices and patient demographics. Our protocol involved monitoring epidural catheter tips for bacterial growth using a standardized removal and culture technique while also recording variables related to patient demographics, surgical context, and catheter placement technique. The data collected will help inform future aseptic practices and strategies to reduce infection rates in postoperative analgesia.

## Materials and methods

This prospective observational study was conducted at Unidade Local de Saúde da Região de Leiria (ULSRL), a secondary care center in Portugal, between November 2022 and March 2023. The study was conducted in accordance with the Declaration of Helsinki, and the study protocol was approved by the Ethics Committee of the study center (approval number: 66/2022). Written informed consent was obtained from all participants or their legal guardians.

Patients eligible for inclusion were those undergoing surgery requiring postoperative epidural analgesia. Exclusion criteria included patients under 18 years of age, catheters used solely for labor analgesia and removed immediately after vaginal delivery, catheters visibly contaminated during removal, those unintentionally dislodged, and catheters removed by a professional outside the acute pain management team.

Epidural catheters were placed using a sterile technique in the operating room immediately before surgery or in the delivery room in obstetric cases where vaginal delivery was planned. The catheter insertion site was prepared with an antiseptic solution (either chlorhexidine or iodopovidone), and all insertions were performed by experienced anesthesiologists in epidural anesthesia. All patients received perioperative prophylactic antibiotics, including cefazolin, ceftriaxone, gentamicin, metronidazole, ciprofloxacin, or ceftazidime, in various combinations. The first dose of antibiotics was administered 60 minutes prior to the surgical incision.

Data were collected on patient characteristics (gender, age, comorbidities, weight, height, and history of infection previous to the neuraxial approach, excluding those involving the neuraxis or the puncture site, which would contraindicate the epidural), surgical details (surgical specialty, regimen, classification of surgical wound), and epidural catheter details (technique, local of placement, level of puncture, number of attempts, type of postoperative analgesia, duration of catheterization). Postoperatively, patients were evaluated for signs and symptoms of local or systemic infection (fever, inflammation, local pain, neurologic abnormalities). The primary endpoint was the incidence of catheter tip colonization, defined by the detection of bacteria through microbiological culture. In addition, we explored potential risk factors for colonization, identified the colonizing organisms and their antibiotic resistance profiles, and assessed signs of local or systemic infection.

Catheter removal was assured by the acute pain management team, which was trained to follow a standardized method to warrant the aseptic collection of the catheter tip, minimizing the potential for sample contamination. To remove the catheter, the operator's hands were washed, and a cap and mask were worn. The patient's skin was disinfected with a 70% alcohol-based solution and allowed to dry. The catheter was then carefully extracted using sterile gloves after hand disinfection. A 3 cm section was cut with sterile scissors and placed in a sterile container for analysis. Upon removal, catheter tips were sent to the microbiology laboratory for culture. Catheters were cultured using a semi-quantitative method, based on the technique described by Maki et al. [10]. Each catheter segment was cultured at 37°C under aerobic conditions for 48 hours, and bacterial identification was carried out using standard methods and criteria. A positive culture was defined as >15 colony-forming units (CFUs). An antibiotic sensitivity test was also conducted whenever the catheter culture was positive.

Statistical analysis was conducted using IBM SPSS Statistics for Windows, Version 29.0.2.0 (Released 2023; IBM Corp., Armonk, New York, United States). Descriptive statistics employed absolute frequencies (n) and relative percentages (%) for categorical variables and means (M) and standard deviations (SD) for continuous variables. The incidence of bacterial colonization was calculated with 95% confidence intervals (CI). To assess the exploratory power of a set of factors on colonization, logistic regressions were used. Initially, univariate logistic regressions were implemented. Subsequently, variables with a p-value of p<0.10 were included in the adjusted analysis. The Hosmer-Lemeshow test was used to evaluate the fit of the data to the logistic model. The significance level considered for rejecting the null hypothesis was 5%.

## Results

The study included 215 patients who underwent epidural analgesia, with their general characteristics presented in Table 1. The majority of the patients were female (165 patients, 76.7%). The average age of the sample was 52.12 years (SD: 19.53 years). The average patient body mass index was 28.72 (SD=5.16).

**Table 1:** General characteristics of the patient sample Results are presented in the format of number (n) and percentage (%) for categorical variables and means (M) and standard deviations (SD) for continuous variables. ASA-PS: American Society of Anesthesiologists physical status

Variable	
Gender	
Female	165 (76.7)
Male	50 (23.3)
Age (years), M (SD)	52.12 (19.53)
Weight (kg), M (SD)	75.29 (13.46)
Height (cm), M (SD)	162.06 (7.39)
ASA-PS, n (%)	
I	6 (2.8)
II	158 (73.5)
III	49 (22.8)
IV	2 (0.9)
Documented infection prior to the neuraxial approach, n (%)	
Yes	4 (1.9)
No	211 (98.1)
Regimen of surgery, n (%)	
Elective	130 (60.5)
Emergent	85 (39.5)
Surgical specialty, n (%)	
General surgery	19 (8.8)
Gynecology	15 (7)
Obstetrics	89 (41.4)
Orthopedics	76 (35.3)
Urology	16 (7.4)
Surgical wound classification, n (%)	
Clean	76 (35.3)
Clean-contaminated	128 (59.5)
Contaminated	11 (5.2)

Regarding surgical specialty, the largest patient group was admitted under obstetrics (89 patients, 41.4%), followed by orthopedics (76 patients, 35.3%). Most surgeries were elective (130 patients, 60.5%), while 85 patients (39.5%) underwent emergency procedures. The most common surgery was cesarean section, accounting for 83 cases (38.6%). More than half of the surgeries were classified as clean-contaminated (128 patients, 59.5%), which included procedures involving the gastrointestinal or genitourinary tract.

The majority of patients were classified as American Society of Anesthesiologists (ASA) physical status II (158 patients, 73.5%). Most patients had no documented infections prior to the neuraxial procedure (211 patients, 98.1%), with the four documented infections being two urinary tract infections and two respiratory tract infections.

Table 2 summarizes the characteristics of the neuraxial technique and postoperative catheter-related outcomes. Regarding the neuraxial approach, 108 patients (50.2%) were submitted to epidural, while 107 patients (49.8%) had a combined spinal-epidural. Most neuraxial interventions occurred in the operating room (157 patients, 73%), and in almost all patients, disinfection was performed with iodopovidone (213 patients, 99.1%). The majority of epidural punctures were performed in the lumbar region (207 patients, 96.3%) with a single successful attempt (141 patients, 65.6%).

**Table 2:** Characteristics of the neuraxial technique and postoperative catheter-related outcomes Results are presented in the format n (%) for categorical variables and means (M) and standard deviations (SD) for continuous variables. The incidence of bacterial colonization was calculated with 95% confidence intervals (CI). Closed-circuit analgesia was given through an elastomeric pump or a patient-controlled epidural analgesia device.

Variable	
Neuraxial approach, n (%)	
Epidural	108 (50.2)
Combined spinal-epidural	107 (49.8)
Physical location of neuraxial technique, n (%)	
Operating room	157 (73)
Delivery room	58 (27)
Puncture level, n (%)	
Lumbar	207 (96.3)
Thoracic	8 (3.7)
Attempts, n (%)	
1	141 (65.6)
2	48 (22.3)
≥3	26 (12.1)
Postoperative analgesia, n (%)	
Manual bolus	85 (39.5)
Closed circuit	130 (60.5)
Signs/symptoms of catheter-related infection, n (%)	
No	208 (96.7)
Yes	7 (3.3)
Positive cultures, n (%, 95% CI)	
No	180 (83.7, 78.8-88.7)
Yes	35 (16.3, 11.3-21.2)

The most common method of administering postoperative analgesia was through a closed circuit, utilizing either an elastomeric pump or a patient-controlled epidural analgesia (130 patients, 60.5%). In contrast, 39.5% of cases (85 patients) received analgesia via manual bolus.

The duration of the catheter was assessed in 142 patients with an average of 2.66 days (SD=0.96, minimum of 0.7 days, and maximum of 6.5 days).

While the epidural catheter was in place, most patients did not present associated symptoms of local or systemic infection (208 patients, 96.7%), and the majority had negative culture results (180 patients, 83.7%). Seven patients (3.3%) exhibited signs of infection, including inflammation at the catheter insertion site, but none was suspicious for epidural space infection (none of them exhibited back pain, root pain, sensory or motor deficit, or fever). The incidence of catheter colonization was 16.3% (95% CI=11.3-21.2%), corresponding to 35 patients.

Among the isolated bacteria, *Staphylococcus epidermidis* was the most common microorganism (13 patients, 37.1%), followed by *Staphylococcus hominis* (nine patients, 25.7%),* Staphylococcus haemolyticus* (four patients, 11.4%), *Staphylococcus capitis* (three patients, 8.6%), and *Staphylococcus aureus* (two patients, 5.7%). *Staphylococcus auricularis, Escherichia coli, Enterococcus faecalis,* and *Proteus mirabilis* were found in 2.9% of cases (one patient each). Of the 35 cases with colonized catheters, 26 (74.3%) exhibited antibiotic resistance, and 17 (48.5%) showed resistance to more than three different antibiotic classes, with oxacillin resistance being the most common, observed in all these cases.

Table 3 presents the results of univariate and adjusted logistic regressions for positive catheter colonization. Exploratory variables included age, gender, elective vs. emergency surgery, number of punctures, and catheter duration. Variables with a p-value of <0.10 in the univariate model were included in the adjusted model. Patients who required two punctures, compared to one, had significantly higher odds of positive colonization (adjusted odds ratio (aOR)=4.52, p=0.033, 95% CI: (1.14, 29.24)). No significant associations were found for the other exploratory variables.

**Table 3:** Univariate and adjusted logistic regression for positive contamination aOR: adjusted odds ratio; CI: confidence intervals; OR: odds ratio; ‡: p<0.10; *: p<0.05

	Univariate		Adjusted		
	OR	P-value	aOR	P-value	95% CI aOR
Age	1.00	0.872	-	-	-
Gender					
Female	1	1	-	-	-
Male	1.22	0.746	-	-	-
Regimen of surgery					
Elective	1	1	-	-	-
Emergent	1.83	0.263	-	-	-
Attempts					
1	1	1	1	1	1
2	3.27	0.036*	4.52	0.033*	1.14-29.24
≥3	0.77	0.807	1.91	0.592	0.18-20.18
Catheter duration	1.77	0.073‡	1.89	0.057‡	0.98-3.64

Finally, an analysis of the location where the epidural was performed (operating room versus delivery room) within the cesarean section group showed no statistically significant association with colonization (p=0.418).

## Discussion

This is the first study in Portugal to assess epidural catheter colonization and infection rates, as well as the antibiotic resistance profiles of the microorganisms colonizing these catheters. Our findings underscore the robustness of current practices while identifying areas for improvement. We observed a colonization rate of 16.3% (95% CI: 11.3-21.2%), consistent with ranges reported in other studies, suggesting that despite aseptic techniques, catheter colonization remains a common issue across healthcare settings [3-9]. This highlights the need for critical review and enhancement of standard aseptic protocols to better control microbial exposure and colonization.

Unlike previous studies that found no link between the number of punctures and colonization, our results show that multiple punctures significantly increase colonization risk [3,4,6]. Specifically, each additional puncture during catheter placement increased the odds of colonization by approximately 4.5 times, emphasizing the importance of minimizing punctures to reduce microbial entry. While the association between catheter duration and colonization risk did not achieve statistical significance, the observed trend suggests that each additional day of catheter use nearly doubles the risk of colonization. Although not definitive, this aligns with findings from Srivastava et al. and Harde et al., emphasizing the need to carefully consider catheter duration to reduce colonization risk [3,4]. Enhanced training or real-time imaging for placement could potentially decrease the need for multiple punctures, directly impacting colonization rates.

Our analysis showed no significant difference in colonization rates between catheters placed in the operating room and the delivery room during cesarean sections. This finding suggests that the physical location where the epidural catheter is inserted may be less important than the strict application of aseptic techniques. Regardless of the setting, maintaining rigorous aseptic protocols, including proper sterilization and handling during catheter insertion, appears to be the key factor in minimizing colonization risks. This highlights the need for consistent training and vigilance in aseptic practices across all hospital environments.

*Staphylococcus epidermidis *was the most common microorganism identified, along with other skin flora, consistent with previous studies [3,4,6,8,9]. Disruption of the skin barrier during catheter placement likely facilitated bacterial entry. While usually commensal, these bacteria can sometimes cause infections [3]. The presence of gastrointestinal and hospital flora suggests potential breaches in aseptic technique, highlighting the need for ongoing staff training to reduce complications [11,12]. The high incidence of multidrug-resistant bacteria in this study reflects local antibiotic resistance issues, stressing the importance of rational antibiotic use and stricter prescription protocols.

Yentur et al. point out that even with optimal aseptic techniques, it is difficult to fully prevent colonization [12]. This suggests that factors such as the environment and the nature of the flora may predispose catheters to colonization, regardless of the precautions taken. Notably, our study highlights the disparity between high catheter colonization rates (16.3%) and the remarkably low incidence of clinical infections, which is aligned with previous studies [7,9]. 

The main limitation of this study is its setting in a secondary hospital, which may not reflect larger or more diverse populations. Another limitation is the potential for sample contamination during catheter removal, despite standardized protocols. Further research should explore why some colonizations lead to infections and investigate other variables affecting colonization and infection rates to inform targeted prevention strategies.

## Conclusions

This study found a 16.3% epidural catheter colonization rate, primarily with *Staphylococcus epidermidis*, and identified multiple punctures and longer catheter duration as potential risk factors. Despite frequent colonization, clinical infections were rare, highlighting the effectiveness of current infection control measures. The presence of multidrug-resistant bacteria emphasizes the need for improved antibiotic stewardship and aseptic practices. Further research is required to better understand the progression from colonization to infection.
